# Interplay of Socioeconomic Status and Supermarket Distance Is Associated with Excess Obesity Risk: A UK Cross-Sectional Study

**DOI:** 10.3390/ijerph14111290

**Published:** 2017-10-25

**Authors:** Thomas Burgoine, Joreintje D. Mackenbach, Jeroen Lakerveld, Nita G. Forouhi, Simon J. Griffin, Søren Brage, Nicholas J. Wareham, Pablo Monsivais

**Affiliations:** 1 UKCRC Centre for Diet and Activity Research (CEDAR), MRC Epidemiology Unit, University of Cambridge School of Clinical Medicine, Box 285 Institute of Metabolic Science, Cambridge Biomedical Campus, Cambridge CB2 0QQ, UK; Nita.Forouhi@mrc-epid.cam.ac.uk (N.G.F.); Simon.Griffin@mrc-epid.cam.ac.uk (S.J.G.); Soren.Brage@mrc-epid.cam.ac.uk (S.B.); Nick.Wareham@mrc-epid.cam.ac.uk (N.J.W.); p.monsivais@wsu.edu (P.M.); 2 Department of Epidemiology and Biostatistics, Amsterdam Public Health Research Institute, VU University Medical Center, P.O. Box 7057, Amsterdam 1007MB, The Netherlands; j.mackenbach@vumc.nl (J.D.M.); je.lakerveld@vumc.nl (J.L.); 3 Department of Public Health and Primary Care, Institute of Public Health, Forvie Site, Robinson Way, University of Cambridge, Cambridge CB2 0SR, UK

**Keywords:** supermarkets, distance, education, body mass index, obesity, interaction

## Abstract

U.S. policy initiatives have sought to improve health through attracting neighborhood supermarket investment. Little evidence exists to suggest that these policies will be effective, in particular where there are socioeconomic barriers to healthy eating. We measured the independent associations and combined interplay of supermarket access and socioeconomic status with obesity. Using data on 9702 UK adults, we employed adjusted regression analyses to estimate measured BMI (kg/m^2^), overweight (25 ≥ BMI < 30) and obesity (≥30), across participants’ highest educational attainment (three groups) and tertiles of street network distance (km) from home location to nearest supermarket. Jointly-classified models estimated combined associations of education and supermarket distance, and relative excess risk due to interaction (RERI). Participants farthest away from their nearest supermarket had higher odds of obesity (OR 1.33, 95% CI: 1.11, 1.58), relative to those living closest. Lower education was also associated with higher odds of obesity. Those least-educated and living farthest away had 3.39 (2.46–4.65) times the odds of being obese, compared to those highest-educated and living closest, with an excess obesity risk (RERI = 0.09); results were similar for overweight. Our results suggest that public health can be improved through planning better access to supermarkets, in combination with interventions to address socioeconomic barriers.

## 1. Introduction

Overweight and obesity are major and increasingly severe public health problems. By 2050, 60% of men and 50% of women are predicted to be obese in the UK [1]. Obesity is associated with increased risk of type 2 diabetes, coronary heart disease and stroke [2]. These comorbidities and others have substantial social and economic implications. Through lost working hours and healthcare costs for example, overweight and obesity are forecast to cost the UK economy £49.9 billion annually by 2050 [1].

Socio-ecological models suggest that overweight and obesity result from a complex interplay of multiple determinants, operating at individual, interpersonal, organisational, environmental, and public policy levels, among others. An important individual-level factor is socioeconomic status. Clear gradients in obesity rates have been observed across markers of socioeconomic status including education, household income and occupational social class [3,4]. For example, recent Health Survey for England data showed 47% lower obesity prevalence among those with degree level qualifications compared to those with no qualifications [5].

Elsewhere, characteristics of neighborhood food environments, including the density, distribution and variety of food stores within our towns and cities, are viewed as important environmental contributors to overweight and obesity. Offering a range of fresh, healthy produce at a variety of price points, access to neighborhood supermarkets may be especially important for supporting healthy dietary behaviours [6] and healthy weight. However, the scientific evidence base regarding the impact of neighborhood supermarket access on weight remains limited and largely equivocal [7,8]. 

Moreover, low socioeconomic status, and low education (perhaps more so than other markers of socioeconomic status such as income or occupational social class), through a number of related psychosocial and behavioural mechanisms including nutrition knowledge [9] and cooking skills [10], potentially confers increased environmental vulnerability, exaggerating the effects of an environment that lacks healthy food retail [11], and resulting in excess overall levels of and inequalities in obesity [12,13]. Although there has been limited empirical research on this topic, access to fast-food outlets has been shown to amplify social inequalities in body weight [14]. While there is no evidence for the existence of “food deserts” in the UK [15], supermarket access does vary spatially and across populations, resulting in population sub-groups who may be more “at risk” of obesity than others. From a public health perspective, the magnitude of this risk needs to be quantified to justify targeted public health intervention.

The purpose of this study was to establish main independent associations between each of supermarket distance and education, with body weight, overweight and obesity in the UK. Our study was further motivated to better understand the magnitude and excess risk of overweight and obesity associated with greatest supermarket distance and lowest education in combination. 

## 2. Materials and Methods

### 2.1. Study Sample

We used data from the Fenland Study, a population-based cohort of adults aged 29–64 years (born 1950–1975) in Cambridgeshire, UK. Cambridgeshire is a county in the East of England, which comprises urban, suburban and rural locations, and includes major cities such as Peterborough and Cambridge. The Fenland Study was conducted by the MRC Epidemiology Unit [16]. Recruitment took place from general practice lists in Cambridge, Ely and Wisbech between 2005 and 2014. Exclusion criteria were pregnancy, previous diabetes diagnosis, inability to walk unaided, psychosis and terminal illness. At the time of this analysis, 11,857 participants had been enrolled into the study (response rate of 27%). Participants completed general questionnaires related to their lifestyle, medical history and home address, and were weighed and measured by trained researchers. All study procedures were approved by the Health Research Authority NRES Committee East of England-Cambridge Central.

### 2.2. Exposure—Supermarket Distance

Participants’ home addresses were mapped by postcode using a geographic information system (ArcGIS 10, ESRI, Redlands, CA, USA). Postcode boundaries in the UK are generally small, containing only 15 individual addresses on average, and therefore they allow for relatively precise, near address-level geocoding to postcode centroids. Data on the locations of food outlets were sourced from 10 local authorities covering the study area in December 2011, and also mapped according to postcode. Local authorities are responsible for public services and facilities within administrative regions across England. Food outlet records are held by local authorities to facilitate routine food hygiene inspections. These records are kept up to date on a rolling basis. New businesses and those ceasing to trade are required by law to notify their local authority [17], who then update (overwrite) their existing records. The accuracy and completeness of this secondary dataset has been shown previously [18], and these data are aligned with other non-commercial and commercial secondary food outlet datasets frequently used in food environments research in terms of validity, according to the results of a recent meta-analysis [19]. Food outlets were classified by type according to a modified version of a previously published classification scheme (Table S1) as: restaurants; convenience stores; cafes; entertainment venues selling food; specialist retailers such as butchers and fishmongers; fast-food outlets; or supermarkets [18]. We classified supermarkets as the seven national chain retailers, who between them command the majority share (89%) of the UK grocery market: Tesco, Sainsbury’s, ASDA, Morrisons (who between them hold 73% of total market share), Waitrose, Aldi and the Co-operative [20]. We focused on these chain retailers as they uniformly stock a variety of fresh, healthy produce, at a range of price points. Other local and independent supermarkets, including frozen food stores that describe themselves as supermarkets, could not be guaranteed to offer similar product lines, and were included in these analyses as convenience stores. Using Ordnance Survey’s MasterMap Integrated Transport Network data, we used ArcGIS Network Analyst to calculate “supermarket distance” (km) from home to the nearest supermarket along the shortest street network route, which was divided into tertiles.

### 2.3. Outcomes—Body Mass Index, Overweight and Obesity

Body mass index (kg/m^2^) was calculated from measured height and weight. We applied World Health Organization cut-offs for overweight (25 ≥ body mass index < 30) and obesity (≥30).

### 2.4. Statistical Analysis

We used multiple linear and multinomial logistic regression models to estimate associations between tertiles of supermarket distance and each of body mass index, odds of overweight and obesity. Covariates were selected a priori, and adjusted models included the following: age, sex, car access (as a proxy for mobility beyond the immediate neighborhood), smoking status, physical activity energy expenditure (kJ/kg/day, estimated from individually calibrated combined heart rate and movement sensing devices (Actiheart, CamNtech, Papworth Everard, Cambridge, UK) worn for up to six days [21], with participants accruing less than 48 h wear time excluded (*n* = 101)), urban/rural status of home address [22], and assessment centre attended. To minimise residual confounding, we adjusted for the total sum of other food outlets (restaurants, convenience stores, cafes, entertainment venues, specialist retailers, fast-food outlets, and other supermarkets) within a 1 mile Euclidean radius buffer of the participant’s nearest supermarket. We also adjusted for highest educational attainment and total household income as indicators of socioeconomic status. Educational attainment groups were: lowest (≤11 years of education), middle (12 to 13 years of education), and highest (>13 years of education). Total gross household income groups were: lowest (<£20,000), middle (£20,000–£40,000), and highest (>£40,000).

To establish independent associations of education with body mass index, odds of overweight and obesity, we used multiple linear and multinomial logistic regression models adjusting for the same set of covariates, but substituting highest educational attainment for supermarket distance. The reference group in these models was those with highest educational attainment.

We explored the magnitude of overweight and obesity risk associated with lowest education and greatest supermarket distance (most “at risk”), relative to those with highest education and lowest supermarket distance (least “at risk”), using an adjusted multinomial logistic regression model. We estimated relative excess risk due to interaction (RERI) for the most “at risk” sub-group. Excess (supra-additive) risk of overweight and obesity was indicated by a combined risk greater than the additive associations of each risk factor in isolation [23], and a corresponding RERI greater than zero. RERI was calculated as follows:

RERI = OR_11_ − OR_10_ − OR_01_ + 1

where ORs are odds ratios for being overweight or obese for those with greatest supermarket distance and lowest education (OR_11_), those with greatest supermarket distance and highest education (OR_10_) and those with lowest supermarket distance and lowest education (OR_01_) [23]. 

In all analyses, we excluded participants with missing data (*n* = 629), as well as those living outside the study area of Cambridgeshire (*n* = 1425), resulting in an analytic sample of 9702. The complete case analytic sample remained representative of the full Fenland Study sample across key variables (Table S2), thereby minimizing the potential for bias. All analyses were conducted using PASW Statistics 21.0 (SPSS Inc., Chicago, IL, USA). 

### 2.5. Ethical Approval

Fenland study volunteers gave written informed consent and all study procedures were approved by the Health Research Authority NRES Committee East of England-Cambridge Central (Ref 04/Q0108/19). All other data analysed was in the public domain.

## 3. Results

### 3.1. Sample Characteristics

Table 1 shows descriptive statistics for the study sample stratified by educational attainment. Mean (SD) age of the participants was 48.1 (7.3) years, with 48.9% of the sample being men. Mean (SD) body mass index was 26.9 (4.7), with 40.3% of the sample being overweight and 21.0% being obese. Mean supermarket distance was 3.8 km, with 52% of the sample living in an urban area. Relative to the most-educated, those least-educated were on average 33% further away from their nearest supermarket. 

Mean body mass index was 2.0 kg/m^2^ greater in those with lowest levels of education compared to those with highest levels of education. A greater proportion of those lowest educated were also overweight or obese. In multivariable adjusted models (Table 1), those with lowest levels of education had a BMI 1.42 kg/m^2^ greater (95% confidence interval (CI): 1.14, 1.70) on average than those most-educated, were 1.68 (95% CI: 1.44, 1.95) times more likely to be overweight and 2.33 (95% CI: 1.93, 2.81) times more likely to be obese.

### 3.2. Independent Associations between Supermarket Distance and Body Mass Index

Greater distance to the nearest supermarket was associated with greater body mass index, with evidence of dose-response associations. In the unadjusted model 1 (Table 2), those living farthest away from their nearest supermarket had an average 1.07 kg/m^2^ greater BMI than those living closest (*p* < 0.001). Adjusting for additional individual and neighborhood level covariates attenuated this association, however those living farthest away remained on average 0.46 kg/m^2^ heavier (*p* < 0.05).

### 3.3. Independent Associations between Supermarket Distance, Overweight and Obesity

Greater distance to the nearest supermarket was associated with greater odds of both overweight and obesity, with evidence of dose-response associations. In the unadjusted model 1 (Table 2), those living farthest away from their nearest supermarket were 1.42 (95% CI: 1.28, 1.59) times more likely to be overweight and 1.83 (95% CI: 1.60, 2.09) times more likely to be obese, than those living closest to their nearest supermarket. Adjusting for additional covariates attenuated these associations, however, those living farthest away remained 1.21 (95% CI: 1.04, 1.40) times more likely to be overweight and 1.33 (95% CI: 1.11, 1.58) times more likely to be obese, than those living closest to their nearest supermarket.

### 3.4. Interplay of Supermarket Distance and Education with Overweight and Obesity

Greater odds of overweight and obesity were associated with farthest distance to the nearest supermarket and lowest educational attainment in combination, in excess of the odds associated with farthest distance or lowest education alone. Table 3 shows adjusted multinomial logistic regression results for each combination of supermarket distance tertile and level of education. Those living farthest away from their nearest supermarket and lowest educated were 2.15 (95% CI: 1.67, 2.77) times more likely to be overweight than those living closest and with highest education. Those living farthest away but most-educated were 1.26 (95% CI: 1.01, 1.57) times more likely to be overweight, while those with lowest education but living closest to their nearest supermarket were 1.49 (95% CI: 1.16, 1.92) times more likely to be overweight. For overweight, relative excess risk due to interaction (RERI) was 0.40. Those living farthest away from their nearest supermarket and lowest educated were 3.39 (95% CI: 2.46, 4.65) times more likely to be obese; odds in excess of the associations of farthest supermarket distance (OR 1.68, 95% CI: 1.24, 2.27) or lowest education (OR 2.62, 95% CI: 1.91, 3.59) in isolation. For obesity, RERI was 0.09.

## 4. Discussion

The purpose of this study was to examine the independent and combined associations of supermarket distance and education with each of body mass index, overweight and obesity, using a large sample of UK adults. In doing so, we contribute to the international research evidence base regarding neighbourhood food environment “effects” on body weight, which to date lacks clarity with regards to the role played by physical access to supermarkets [7,8]. We found that greater supermarket distance was independently associated with higher body mass index and odds of both overweight and obesity, with evidence of dose-response relationships throughout. We also showed independent associations of lower educational attainment with each of these outcomes. 

Importantly, the combined associations of greatest supermarket distance and lowest education with likelihood of both overweight and obesity were in excess of the associations between these outcomes and the presence of only one risk factor (greatest supermarket distance or lowest education), suggesting an additive interaction in this population sub-group. Furthermore, the magnitude of this combined association, which resulted in a threefold greater likelihood of obesity, provides cause for public health concern, particularly as those least educated were the most disadvantaged in terms of supermarket access in this study. Our findings are consistent with international studies that have observed similar compound deprivation effects associated with fast-food [14], and green space access [24].

### 4.1. Possible Mechanisms

Established behavioural, psychosocial and economic attributes, which have been linked to socioeconomic gradients in obesity, may confer increased environmental vulnerability, therefore exaggerating the effects of low supermarket access [12,25]. For example, in areas poorly served by supermarkets, consumers may rely more heavily on foods prepared for them outside of the home [26], for example from fast-food outlets. Fast food outlets typically sell large portions of less healthy food than can be prepared at home [27], and increased patronage of such outlets has been associated with weight gain over time [28]. Within these outlets, the price sensitivity of low income, low-socioeconomic status consumers may result in the selection of energy-dense products that are perceived to offer value for money [29].

Evidence also suggests that individuals are often compelled in areas lacking supermarket access to rely more heavily on convenience stores for their grocery shopping [26]. Convenience stores are typically more expensive than supermarkets, offering more limited product lines [30], while qualitative reports suggest that healthy foods are also more hidden, often among marketing and promotional materials for less healthy foods in particular [26]. These in-store attributes may especially disadvantage groups of low socioeconomic status, who spend less per calorie than any other social group [31]; perceive healthy food to be expensive [32]; may be less equipped to cook healthy meals from scratch from a limited grocery selection [10]; may be more susceptible to unhealthy food marketing and unhealthy product placement [33]; and who may not value their own health sufficiently enough to “hunt” for healthy options in a within-store environment that encourages unhealthy purchases [26]. Moreover, in the absence of accessible supermarkets, reliance on these other types of food outlets near to home may be further exaggerated in groups of low socioeconomic status through unemployment [34], low levels of car access [35] and restricted time budgets [36], which serve to limit and constrain daily mobility. In a UK study, the median distance to a participant’s main grocery store was 63% greater among those with car access as opposed to no car access [37].

A limited number of government policy programmes in the U.S. have already sought to improve local fresh food retail and therefore health [38,39], by specifically working to attract neighbourhood supermarket investment. These policies have been referred to as a “supermarket solution”, yet the existing evidence base does not wholly support their implementation. Our findings suggest that such policies may be effective, and that neighbourhood supermarket access represents a modifiable risk factor for obesity that could be leveraged to reduce both present and future population-level obesity burdens. However, further research is required, as our results stress a second important point of intervention at the level of the individual in order to maximise the success of any new neighbourhood supermarket [26]. While individual level interventions such as those targeting improvements in cooking skills have historically been problematised for relying heavily on individual agency alone for their success [40], structural barriers to intervention uptake are often poorly understood and healthy dietary behaviours might only be sustained within a supportive neighborhood food environment. This hypothesis is supported by the results of a recent U.S. study, where the effects of a weight loss intervention for those with metabolic syndrome were greatest among participants with the best neighborhood grocery store access [41].

Although more research is required to understand the combined effects of interventions at multiple levels to improve diet and health, the identification of those in the most “at risk” population sub-group will also be a major challenge. Supermarket distance in this Cambridgeshire study was highly variable, with distances to the nearest supermarket ranging from zero to 15 kilometres. Understanding and identifying nationwide address-level differences in supermarket access, in conjunction with socioeconomic data, and tracking these differences over time in response to neighborhood supermarket interventions, would represent important first steps in addressing extreme access disparities and monitoring their reduction.

### 4.2. Strengths and Limitations

Supermarket distance exposure misclassification could have resulted from the assumption that study participants would shop at the closest supermarket to their home. Although supermarket distance was not constrained to arbitrarily defined neighborhood boundaries, we had no information on where or at which supermarket participants actually shopped for food. A U.S. study showed that only one in seven food shoppers chose to patronise the supermarket closest to their home [42]. However, this ‘uncertain geographic context problem’ is not limited to this study [43]. Furthermore, while supermarkets may be accessed from non-home locations, previous UK research highlighted proximity to home as a major contributor to supermarket choice [37], while 92% of shoppers in one U.S. study reported leaving from home to conduct their grocery shopping [44]. Although our local authority food outlet location data was not ground-truthed for locational accuracy, we have shown previously that this data represents the most accurate of its kind in the UK [18], and is comparable in terms of completeness to other commonly used sources of data in food environment studies [19], minimising further potential risk of exposure misclassification. Temporal mismatch may have resulted in misclassification as our exposures (supermarket locations) and outcomes (body mass index, overweight, obesity) were measured at different time points (2011 and 2005–2014, respectively). However, growth in the UK supermarket sector has historically been slow, with evidence to suggest that new supermarkets co-locate alongside existing supermarkets [45], which would only marginally affect our proximity estimates. Moreover, despite an increase in the overall number of supermarkets across the Fenland Study region over an extended time period (2011–2017), further quantitative analysis (Table S3) shows clear temporal stability in our primary exposure estimates (supermarket proximity from the home locations of Fenland Study participants), resulting in minimal exposure misclassification. On this basis, we presume that supermarket proximity would have remained similarly stable for Fenland Study participants from 2005 to 2014. We also contend that exposure misclassification risk is further reduced through operationalizing supermarket proximity in tertiles, with significant temporal stability also evident in this metric over six years (Table S3). We used highest education as our marker of socioeconomic status, but could have focused on household income, which is often imperfectly correlated with education [46]. Although we included household income as a covariate in all of our models, it is possible that our results could be sensitive to the selection of this socioeconomic marker. Our study was based on observational, cross-sectional data, which limits inference on causal relationships. Yet, our study makes an important and novel contribution to an evidence base that is currently limited, worldwide but especially in the UK, regardless of the study design employed. Finally, our study utilised a representative sample of the Cambridgeshire population that was more educated and less ethnically diverse than the wider UK. While this may limit generalisability to an extent, the population of Cambridgeshire is not unique in a national or international context, ensuring relevance of our findings elsewhere.

## 5. Conclusions

This study used a large and unique UK dataset, containing both individual level geographic and well characterised socioeconomic information, combined with accurate neighborhood environment data, to examine independent and combined associations of geographic supermarket access and educational attainment with multiple, objectively measured adiposity outcomes. We showed strong associations between each of greater supermarket distance and lower education, with higher body weight, greater odds of overweight and obesity, and with evidence of dose-response relationships. We also demonstrated an excess risk of overweight and obesity associated with greatest supermarket distance and lowest education, above and beyond the level of risk associated with experiencing each of these risk factors in isolation, which is a matter of public health concern. We provide novel UK evidence that reductions in obesity would be best achieved through improving supermarket access in combination with interventions to address socioeconomic barriers.

## Figures and Tables

**Table 1:** Characteristics of participants in the Fenland Study sample, Cambridgeshire, UK, by educational attainment (*n* = 9702).

Variable	Educational Attainment ^a^			All (*n* = 9702)
	Highest (*n* = 3157)	Middle (*n* = 4565)	Lowest (*n* = 1980)	
Age, years	47.1 (7.6)	48.3 (7.1)	49.1 (7.0)	48.1 (7.3)
Men (*n* (%) of participants)	1603 (50.8)	2301 (50.4)	843 (42.6)	4747 (48.9)
Physical activity energy expenditure, kJ/kg/day	53.1 (20.1)	55.1 (22.5)	54.8 (24.0)	54.4 (22.1)
Household income, >£40,000 (*n* (%) of participants)	2360 (74.8)	2090 (45.8)	585 (29.5)	5035 (51.9)
Car access (*n* (%) of participants)	2861 (90.6)	4393 (96.2)	1865 (94.2)	9119 (94.0)
Current smoker (*n* (%) of participants)	199 (6.3)	604 (13.2)	347 (17.5)	1150 (11.9)
Distance to nearest supermarket, km	3.0 (3.4)	4.2 (3.7)	4.0 (3.6)	3.8 (3.6)
Food outlets within a 1 mile Euclidean buffer of the nearest supermarket, *n*	134.6 (119.7)	79.8 (69.6)	76.9 (60.2)	97.0 (91.5)
Urban (*n* (%) of participants)	1885 (59.7)	2147 (47.0)	1011 (51.1)	5043 (52.0)
Assessment centre, Cambridge (*n* (%) of participants)	1933 (61.2)	1135 (24.9)	372 (18.8)	3440 (35.5)
*Crude weight status outcomes*				
Body mass index, kg/m^2^	25.7 (4.3)	27.3 (4.8)	27.7 (4.9)	26.9 (4.7)
Overweight, 25 kg/m^2^ ≥ BMI < 30 kg/m^2^ (*n* (%) of participants)	1178 (37.3)	1897 (41.6)	833 (42.1)	3908 (40.3)
Obese, BMI ≥ 30 kg/m^2^ (*n* (%) of participants)	414 (13.1)	1110 (24.3)	514 (26.0)	2038 (21.0)
*Adjusted weight status outcomes ^b^*				
Body mass index (kg/m^2^), β (95% CI)	ref	1.14 (0.91, 1.36) **^,c^	1.42 (1.14, 1.70) **^,c^	-
Overweight (25 kg/m^2^ ≥ BMI < 30 kg/m^2^), OR (95% CI)	ref	1.46 (1.29, 1.64) **^,d^	1.68 (1.44, 1.95) **^,d^	-
Obese (BMI ≥ 30 kg/m^2^), OR (95% CI)	ref	2.07 (1.78, 2.41) **^,d^	2.33 (1.93, 2.81) **^,d^	-

Data are mean (standard deviation) unless otherwise stated; ** *p* < 0.001; ^a^ Educational attainment, three groups: Lowest, ≤11 years of education; Middle, 12 to 13 years of education; Highest, >13 years of education; ^b^ Adjusted for age, sex, household income, car access, smoking status, physical activity energy expenditure, assessment centre attended, urban/rural status of home address, supermarket proximity and exposure to other food outlets within a 1 mile Euclidean radius buffer of the nearest supermarket; ^c^ Results from a multiple linear regression model, estimating β coefficients for difference in body mass index with 95% confidence intervals (CIs), relative to those highest educated (ref); ^d^ Results from a single multinomial logistic regression model, estimating odds ratios (ORs) for overweight and obesity with 95% confidence intervals, relative to those highest educated (ref).

**Table 2:** Associations of tertiles of distance to the nearest supermarket with body mass index (estimated using a multiple linear regression model), overweight and obesity (estimated using a single multinomial logistic regression model), in the Fenland Study sample (*n* = 9702).

**BMI**	**Tertile (Range)**	**Model 1 ^a^**		**Model 2 ^b^**		**Model 3 ^c^**	
		**β**	**95% CI**	**β**	**95% CI**	**β**	**95% CI**
Distance to nearest supermarket tertile ^d^	T1 (0.0–1.1 km)	ref		ref		ref	
	T2 (1.1–4.9 km)	0.84 **	0.61, 1.07	0.26 *	0.03, 0.49	0.23	0.00, 0.46
	T3 (4.9–15.1 km)	1.07 **	0.84, 1.30	0.46 **	0.24, 0.69	0.46 *	0.18, 0.74
**Overweight**	**Tertile (Range)**	**Model 1 ^a^**		**Model 2 ^b^**		**Model 3 ^c^**	
		**OR**	**95% CI**	**OR**	**95% CI**	**OR**	**95% CI**
Distance to nearest supermarket tertile ^d^	T1 (0.0–1.1 km)	ref		ref		ref	
	T2 (1.1–4.9 km)	1.30 **	1.17, 1.45	1.14 *	1.01, 1.29	1.12	1.00, 1.27
	T3 (4.9–15.1 km)	1.42 **	1.28, 1.59	1.22 *	1.08, 1.37	1.21 *	1.04, 1.40
**Obesity**	**Tertile (Range)**	**Model 1 ^a^**		**Model 2 ^b^**		**Model 3 ^c^**	
		**OR**	**95% CI**	**OR**	**95% CI**	**OR**	**95% CI**
Distance to nearest supermarket tertile ^d^	T1 (0.0–1.1 km)	ref		ref		ref	
	T2 (1.1–4.9 km)	1.57 **	1.37, 1.80	1.18 *	1.02, 1.38	1.16	1.00, 1.35
	T3 (4.9–15.1 km)	1.83 **	1.60, 2.09	1.37 **	1.18, 1.59	1.33 *	1.11, 1.58

* *p* < 0.05; ** *p* < 0.001; ^a^ Model 1 is an unadjusted model; ^b^ Model 2 adjusts for age, sex, household income, car access, highest educational attainment, smoking status, physical activity energy expenditure and assessment centre attended (individual level covariates); ^c^ Model 3 additionally adjusts for urban/rural status of home address and exposure to other food outlets within a 1 mile Euclidean radius buffer of the nearest supermarket; ^d^ T1 = tertile with shortest distance to the nearest supermarket–T3 = tertile with longest distance to the nearest supermarket.

**Table 3:** Additive interaction between supermarket distance and educational attainment on the likelihood of being overweight or obese, modelled using a single multinomial logistic regression model in the Fenland Study sample (*n* = 9702).

	Distance to Nearest Supermarket Tertile ^a^					
	T1 (0.0–1.1 km)		T2 (1.1–4.9 km)		T3 (4.9–15.1 km)	
**Educational Attainment ^b^**	**Overweight/Normal Weight (*n*)**	**OR (95% CI)**	**Overweight/Normal Weight (*n*)**	**OR (95% CI)**	**Overweight/Normal Weight (*n*)**	**OR (95% CI)**
Highest	541/793	ref ^c^	315/427	1.00 (0.82, 1.21);	322/345	1.26 (1.01, 1.57);
				*p* = 0.96 ^d^		*p* = 0.05 ^d^
Middle	509/483	1.44 (1.19, 1.73);	677/511	1.71 (1.42, 2.06);	711/564	1.59 (1.30, 1.94);
		*p* < 0.001 ^d^		*p* < 0.001 ^d^		*p* < 0.001 ^d^
Lowest	196/185	1.49 (1.16, 1.92);	326/249	1.80 (1.44, 2.26);	311/199	2.15 (1.67, 2.77);
		*p* = 0.002 ^d^		*p* < 0.001 ^d^		*p* < 0.001 ^d^
**Educational Attainment ^b^**	**Obese/Normal Weight (*n*)**	**OR (95% CI)**	**Obese/Normal Weight (*n*)**	**OR (95% CI)**	**Obese/Normal Weight (*n*)**	**OR (95% CI)**
Highest	152/793	ref ^c^	124/427	1.20 (0.90, 1.59);	138/345	1.68 (1.24, 2.27);
				*p* = 0.21 ^d^		*p* = 0.001 ^d^
Middle	276/483	2.31 (1.80, 2.97);	384/511	2.74 (2.13, 3.51);	450/564	2.83 (2.17, 3.70);
		*p* < 0.001 ^d^		*p* < 0.001 ^d^		*p* < 0.001 ^d^
Lowest	129/185	2.62 (1.91, 3.59);	202/249	2.91 (2.18, 3.88);	183/199	3.39 (2.46, 4.65);
		*p* < 0.001 ^d^		*p* < 0.001 ^d^		*p* < 0.001 ^d^

Measure of interaction on an additive scale (RERI) for overweight = 0.40; for obesity = 0.09. RERI scores >0 suggest positive interaction and departure from additivity. ORs are adjusted for age, sex, household income, smoking status, car access, physical activity energy expenditure, assessment centre attended, urban/rural status of home address and exposure to other food outlets within a 1 mile Euclidean radius buffer of the nearest supermarket. ^a^ T1 = tertile with shortest distance to the nearest supermarket–T3 = tertile with longest distance to the nearest supermarket; ^b^ Educational attainment, three groups: Lowest, ≤11 years of education; Middle, 12 to 13 years of education; Highest, >13 years of education; ^c^ Single reference group; those highest educated and with shortest distance to the nearest supermarket; ^d^ ORs and *p* values relative to single reference group (ref).

## Supplementary Materials

The following are available online at www.mdpi.com/1660-4601/14/11/1290/s1, Table S1: Details of the food outlet classification system used, according to primary retail function, with UK examples of chains and types (where applicable). This seven point food outlet classification system was derived from a more detailed 21-point schema published by Lake et al. (2010), Table S2: Fenland Study sample (*n* = 11,857) and Fenland Study analytic sample (*n* = 9702) demographic comparisons, Table S3: Evidence of temporal stability in our primary exposure (supermarket proximity (km, tertiles) from the home locations of 9702 Fenland Study participants), 2011–2017.
