# Supplementary material for: Interplay of Socioeconomic Status and Supermarket Distance Is Associated with Excess Obesity Risk: A UK Cross-Sectional Study
Source: Int J Environ Res Public Health. 2017 Oct 25;14(11):1290. doi: 10.3390/ijerph14111290 (PMC5707929; doi:10.3390/ijerph14111290)
Supplement: Supplementary file 1 [file ijerph-14-01290-s001.pdf]

Supplementary Materials: Interplay of Socioeconomic Status and Supermarket Distance Is Associated with Excess Obesity Risk: A UK Cross-Sectional Study

Thomas Burgoine, Joreintje D. Mackenbach, Jeroen Lakerveld, Nita G. Forouhi, Simon J. Griffin, Søren Brage, Nicholas J. Wareham and Pablo Monsivais

Table S1. Details of the food outlet classification system used, according to primary retail function, with UK examples of chains and types (where applicable). This seven point food outlet classification system was derived from a more detailed 21-point schema published by Lake *et al* (2010) [1].

Type of Outlet	Common Defining Characteristics; Chain and Type Examples
Restaurant	• Food orders are taken by wait staff<br>• Food served to be consumed on the premises<br>• Food delivered to the table<br>• Food paid for after eating
	Type: by cuisine, pizza, burger, fine dining; *by origin*, British, Chinese, Italian, Indian, American
	Chains: Pizza Express, Gourmet Burger Kitchen, Pizza Hut, Nando's
Convenience store	• Sells limited range of groceries, newspapers/magazines, snacks, drinks, lottery, tobacco and alcohol products<br>• Often have extended opening hours<br>• Usually small in size
	Type: convenience stores, newsagents, frozen, discount<br>*Chains:* Londis, SPAR, Nisa, Premier Stores, Heron Foods, Poundland
Supermarket	• Departmentalised, self-service mutiple chain store selling a wide range of groceries and household goods
	Type: large, small
	Chains: Tesco, Sainsbury's, ASDA, Morrisons, the Co-operative, Waitrose, Aldi, plus small-formats e.g., Tesco Express, Sainsbury's Local
Fast-food	• Hot food sold<br>• Food ordered and paid for at the till<br>• No wait staff<br>• No or limited, informal seating options for dining in
	Type: by cuisine, pizza, burger, fried chicken, fish and chips, chain bakery; *by origin*, British, Chinese, Italian, Indian, American
	Chains: McDonalds, Burger King, KFC, Subway, Greggs, Domino's Pizza, Papa John's, Pizza Hut Delivery
Entertainment venue	• Primarily visited for entertainment, with food service a secondary function
	Type: cinema, bowling, theatre, sports and music venues, amusement arcades, health clubs, leisure centres
Café	• Predominantly sell hot beverages, snacks and/or light meals, including sandwiches<br>• Consumption possible on or off the premises<br>• Limited or informal seating area
	Type: coffee shop, tea room, sandwich shop
	Chains: Starbucks, Costa Coffee, Caffe Nero
Specialist	• Stores selling a specific range of produce
	Type: butchers, bakers, delicatessens, fishmongers, confectioners, greengrocers, organic, oriental, artisan food stores
	Chains: few, mostly independent

Table S2. Fenland Study sample (n = 11,857) and Fenland Study analytic sample (n = 9702) demographic comparisons.

Variable	Full Fenland Study Sample (n = 11,857)				Fenland Study Analytic Sample (n = 9702)			
	**Mean (SD)**	**Range**	**Median**	**IQR**	**Mean (SD)**	**Range**	**Median**	**IQR**
Age	48.4 (7.5)	29.4–64.0	48.6	42.4–54.5	48.1 (7.3)	29.4–64.0	48.2	42.3–54.0
BMI	26.9 (4.8)	14.5–59.9	26.2	23.6–29.4	26.9 (4.7)	14.5–58.7	26.2	23.6–29.3
Sex, men (%)	46.2				48.9			
Household Income, >£40,000 (%)	50.4				51.9			

Table S3. Evidence of temporal stability in our primary exposure (supermarket proximity (km, tertiles) from the home locations of 9702 Fenland Study participants), 2011–2017 [a].

Supermarket Proximity	Year of Origin of Supermarket Data	
	**2011**	**2017**
Mean (SD) distance, km	3.8 (3.6)	3.6 (3.5)
Minimum distance, km	0.0	0.0
Maximum distance, km	15.1	15.1
Correlation co-efficients:		
r_P, km [b]	0.916 **	
r_S, tertile [c]	0.913 **	

** $p < 0.001$. [a] Introduction: In this study, food outlet data were collected at one time point in 2011, and Fenland Study participants were sampled from 2005–2014. Changes in the food environment across the data collection period may have resulted in the misclassification of supermarket proximity (our primary exposure) for some participants. Growth in the UK supermarket sector has historically been slow, with evidence that new supermarkets co-locate alongside existing supermarkets [41], which would suggest our proximity estimates would be marginally affected. However, we wanted to quantify the extent to which changes in numbers of supermarkets across the study region, *over an extended period of time*, would be reflected in this primary exposure. Method: we collected data on the locations of the same chain supermarkets (Tesco, Sainsbury's, ASDA, Morrisons, Waitrose, Aldi and the Co-operative) from the same sources (local authorities) in March 2017, six calendar years after the data used in this study were collected. Distance (km) and tertiles of distance from the home locations of Fenland Study participants to their nearest supermarket using 2017 supermarket data were calculated, and compared to 2011 distances using descriptive statistics and correlation analyses. Results: numbers of supermarkets had increased throughout the Fenland Study region over this time, from 134 to 162 (20.1% increase). Mean, minimum and maximum distances were very similar or identical. Correlation co-efficients for distance (continuous, km) and tertiles of distance were 0.916 and 0.913, respectively (both $p < 0.001$). Conclusions: despite change in the overall number of supermarkets, results indicated temporal stability in supermarket proximity for Fenland Study participants over six years. While this analysis of change in our primary exposure does not cover the entirety of the Fenland Study data collection period (2005–2014), it provides clear evidence of temporal stability in these exposure estimates over time, which in direct relation to our sample, resulted in minimal exposure misclassification; [b] Pearson's correlation co-efficient (r_P) for 2011 and 2017 distances to nearest supermarket (km); [c] Spearman's rank correlation co-efficient (r_S) for 2011 and 2017 tertiles of distance to nearest supermarket.

References

1. Lake, A.A.; Burgoine, T.; Greenhalgh, F.; Stamp, E.; Tyrrell, R. The foodscape: Classification and field validation of secondary data sources. *Health Place* **2010**, *16*, 666–673.

© 2017 by the authors; licensee MDPI, Basel, Switzerland. This article is an open access article distributed under the terms and conditions of the Creative Commons by Attribution (CC-BY) license (http://creativecommons.org/licenses/by/4.0/).
